# An Open-Source Deep Learning Algorithm for Efficient and Fully Automatic Analysis of the Choroid in Optical Coherence Tomography

**DOI:** 10.1167/tvst.12.11.27

**Published:** 2023-11-21

**Authors:** Jamie Burke, Justin Engelmann, Charlene Hamid, Megan Reid-Schachter, Tom Pearson, Dan Pugh, Neeraj Dhaun, Amos Storkey, Stuart King, Tom J. MacGillivray, Miguel O. Bernabeu, Ian J. C. MacCormick

**Affiliations:** 1 School of Mathematics, University of Edinburgh, Edinburgh, UK; 2 School of Informatics, University of Edinburgh, Edinburgh, UK; 3 Centre for Medical Informatics, University of Edinburgh, Edinburgh, UK; 4 Clinical Research Facility and Imaging, University of Edinburgh, Edinburgh, UK; 5 University Hospital Wales, NHS Wales, Cardiff, Wales, UK; 6 British Heart Foundation Centre for Cardiovascular Science, University of Edinburgh, Edinburgh, UK; 7 Institute for Adaptive and Neural Computation, School of Informatics, University of Edinburgh, Edinburgh, UK; 8 Centre for Clinical Brain Sciences, University of Edinburgh, Edinburgh, UK; 9 The Bayes Centre, University of Edinburgh, Edinburgh, UK; 10 Centre for Inflammation Research, The Queen's Medical Research Institute, University of Edinburgh, Edinburgh, UK

**Keywords:** choroid, optical coherence tomography, deep learning, segmentation

## Abstract

**Purpose:**

To develop an open-source, fully automatic deep learning algorithm, DeepGPET, for choroid region segmentation in optical coherence tomography (OCT) data.

**Methods:**

We used a dataset of 715 OCT B-scans (82 subjects, 115 eyes) from three clinical studies related to systemic disease. Ground-truth segmentations were generated using a clinically validated, semiautomatic choroid segmentation method, Gaussian Process Edge Tracing (GPET). We finetuned a U-Net with the MobileNetV3 backbone pretrained on ImageNet. Standard segmentation agreement metrics, as well as derived measures of choroidal thickness and area, were used to evaluate DeepGPET, alongside qualitative evaluation from a clinical ophthalmologist.

**Results:**

DeepGPET achieved excellent agreement with GPET on data from three clinical studies (AUC = 0.9994, Dice = 0.9664; Pearson correlation = 0.8908 for choroidal thickness and 0.9082 for choroidal area), while reducing the mean processing time per image on a standard laptop CPU from 34.49 ± 15.09 seconds using GPET to 1.25 ± 0.10 seconds using DeepGPET. Both methods performed similarly according to a clinical ophthalmologist who qualitatively judged a subset of segmentations by GPET and DeepGPET, based on smoothness and accuracy of segmentations.

**Conclusions:**

DeepGPET, a fully automatic, open-source algorithm for choroidal segmentation, will enable researchers to efficiently extract choroidal measurements, even for large datasets. As no manual interventions are required, DeepGPET is less subjective than semiautomatic methods and could be deployed in clinical practice without requiring a trained operator.

**Translational Relevance:**

DeepGPET addresses the lack of open-source, fully automatic, and clinically relevant choroid segmentation algorithms, and its subsequent public release will facilitate future choroidal research in both ophthalmology and wider systemic health.

## Introduction

The retinal choroid is a complex, extensively interconnected vessel network positioned between the retina and the sclera. The choroid holds the majority of the vasculature in the eye and plays a pivotal role in nourishing the retina. Optical coherence tomography (OCT) is an ocular imaging modality that uses low-coherence light to construct a three-dimensional map of chorioretinal structures at the back of the eye. Standard OCT imaging does not visualize the deeper choroidal tissue well, as the tissue sits beneath the hyperreflective retinal pigment epithelium layer of the retina. Enhanced depth imaging OCT (EDI-OCT) overcomes this problem and offers improved visualization of the choroid, thus providing a unique window into the microvascular network that not only resides closest to the brain embryologically but also carries the highest volumetric flow per unit tissue weight compared to any other organ in the body.

Since the advent of OCT, interest in the role played by the choroid in systemic health has been growing,^1^ as non-invasive imaging of the choroidal microvasculature may provide a novel location to detect systemic, microvascular changes early. Indeed, changes in choroidal blood flow, thickness, and other markers have been shown to correspond with patient health such as choroidal thickness in chronic kidney disease^2^ and choroidal area and vascularity in Alzheimer's dementia.^3^

Quantification of the choroid in EDI-OCT imaging requires segmentation of the choroidal space. However, this is a more difficult problem than retinal layer segmentation due to poor signal penetration from the device—and thus lower signal-to-noise ratio—and shadows cast by superficial retinal vessels and choroidal stroma tissue. This results in poor intra- and interrater agreement even with manual segmentation by experienced clinicians, and manual segmentation is too labor intensive and subjective to be practical for analyzing large-scale datasets.

Semiautomated algorithms improve on this slightly but are typically multistage procedures, requiring traditional image processing techniques to prepare the images for downstream segmentation.^4^ Methods based on graph theory such as Dijkstra's algorithm^5^^,^^6^ or graph cut,^7^ as well as on statistical techniques including level sets,^8^^,^^9^ contour evolution,^10^ and Gaussian mixture models,^11^ have been proposed previously. Concurrently, deep learning (DL)-based approaches have emerged. Chen et al.^12^ used a DL model for choroid layer segmentation but with traditional contour tracing as a postprocessing step. Other DL-based approaches, too, combine traditional image processing techniques as pre- or postprocessing steps,^13^^–^^15^ whereas others are fully DL based,^16^^,^^17^ the latter of which is in a similar vein to the proposed method. More recently, DL has been used to distill existing semiautomatic traditional image processing pipelines into a fully automatic method.^18^

Gaussian Process Edge Tracing (GPET), based on Bayesian machine learning,^19^ is a particularly promising method for choroid layer segmentation that has been clinically and quantitatively validated.^20^ Gaussian process regression is used to model the upper and lower boundaries of the choroid from OCT scans. For each boundary, a recursive Bayesian scheme is employed to iteratively detect boundary pixels based on the image gradient and the distribution of candidate boundaries by the Gaussian process regressor. However, GPET is semiautomatic and thus requires time-consuming manual interventions by specifically trained personnel, which introduces subjectivity and limits the potential for analyzing larger datasets or deploying GPET into clinical practice.

There are currently no accessible, open-source algorithms for fully automatic choroidal segmentation. All available algorithms fall into one of three categories: First, semiautomatic methods^21^^,^^22^ that require human supervision and training and introduce subjectivity. Second, fully automatic DL-based methods that are not openly accessible, either providing only the code but not the trained model necessary to use the method^23^ or not providing any access at the time of writing.^24^ Third, fully automatic algorithms comprising many steps that require a good understanding of image processing techniques and a license for proprietary software (MATLAB; MathWorks, Natick, MA).^25^

**Figure 1.**
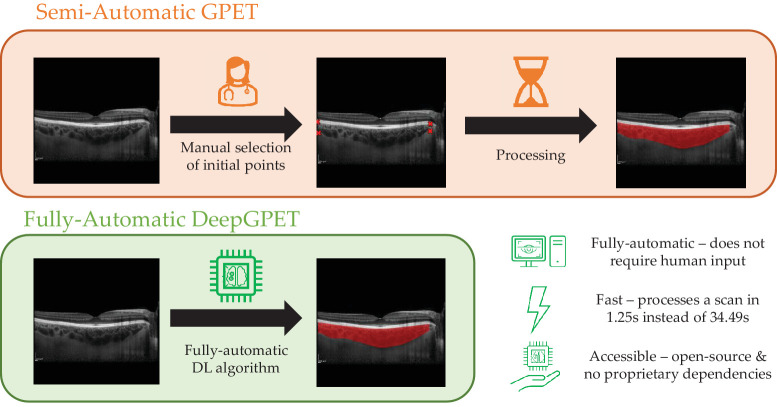
Comparison between the semi-automatic GPET^19^^,^^20^ (*top*) and fully automatic DeepGPET (*bottom*).

We aimed to develop and release an open-source, raw image-to-measurement, fully automatic method for choroid region segmentation that can be easily used without special training and does not require licenses for proprietary software (Fig. 1). Importantly, we intend not only to make our method available to the research community but also to do so in a frictionless way that allows other researchers to download and use our method without seeking our approval. We distill GPET into a DL algorithm, DeepGPET, which can process images without supervision in a fraction of the time, permitting analysis of large-scale datasets and potential deployment into clinical care and research practice without prior training in image processing. The code and model weights for DeepGPET are available at https://github.com/jaburke166/deepgpet.

## Methods

### Study Population

We used 715 OCT B-scans belonging to 82 subjects from three studies: (1) OCTANE,^26^ a study looking at renal function and impairment in chronic kidney disease patients; (2) i-Test, a study recruiting pregnant women of any gestation or those who have delivered a baby within 6 months, including controls and individuals at high risk of complications; and (3) normative data from 30 healthy volunteers as a control group.^27^ All studies conformed to the tenets of the Declaration of Helsinki and received relevant ethical approval and informed consent from all subjects. Table 1 provides an overview of basic population characteristics and number of subjects and images for these studies. Supplementary Figure S1 presents boxplot distributions of choroidal thickness and area for the three datasets used to build DeepGPET, and Table 1 presents tabular mean and standard deviation values.

**Table 1.** Overview of Population Characteristics

	OCTANE	i-Test	Control	Total
Subjects, *n*	47	5	30	82
Male/female, *n*	24/23	0/5	20/10	44/38
Right/left eyes, *n*	47/0	5/5	29/29	81/34
Age (y), mean (SD)	48.8 (12.9)	34.4 (3.4)	49.1 (7.0)	48.0 (11.2)
Machine	Standard	FLEX	Standard	Both
Horizontal/vertical scans, *n*	166/0	16/16	57/54	239/70
Volume scans, *n*	174	186	46	406
Total B-scans, *n*	340	218	157	715

Two Heidelberg Engineering (Heidelberg, Germany) spectral-domain OCT SPECTRALIS devices were used for image acquisition: the Standard Module (OCT1 system) and FLEX Module (OCT2 system). The FLEX is a portable version that enables imaging of patients in a ward environment. Both machines image a 30° region (8.7 mm), generating a macular, cross-sectional OCT B-scan at a resolution of 768 × 768 pixels. Notably, 14% of the OCT B-scans were scanned without EDI mode activated (non-EDI) and thus presented more challenging images with lower signal-to-noise ratios in the choroidal part of the OCT. Horizontal line and vertical scans were centered at the fovea with active eye tracking using an Automatic Real Time (ART) value of 100. Posterior pole macular scans covered a 30° × 25° region using EDI mode.

We split the data into an approximately 85:8:7 split among training (603 B-scans, 66 subjects), validation (58 B-scans, 9 subjects), and test sets (54 B-scans, 7 subjects). When splitting the data, we did so at the patient level; that is, each subject's OCT images were present in only one set and they were selected so that each set had proportionally equal amounts of scan types (EDI/non-EDI) to best represent image quality. See Supplementary Table S1 for an overview of basic population and imaging characteristics for each set.

### DeepGPET

As the ground truths are based on GPET, DeepGPET can be can be seen as a more efficient, fully automatic and distilled version of GPET. Our approach was to fine-tune a U-Net^28^ with a MobileNetV3^29^ backbone pretrained on ImageNet for 60 epochs with batch size 16 using AdamW^30^ (learning rate [LR] = 10^−3^; β_1_ = 0.9; β_2_ = 0.999; weight decay = 10^−2^). After epoch 30, we maintained an exponential moving average of model weights which we then used as our final model. We used the following data augmentations: brightness and contrast changes, horizontal flipping, and simulated OCT speckle noise by applying Gaussian noise followed by multiplicative noise (all *P* = 0.5), and Gaussian blur and random affine transforms (both *P* = 0.25). To reduce memory load, we cropped the black space above and below the OCT B-scan and processed the images at a resolution of 544 × 768 pixels. Images were standardized by subtracting 0.1 and dividing by 0.2, and no further preprocessing was done. We used Python 3.11, PyTorch 2.0, Segmentation Models PyTorch,^31^ and the timm library.^32^

### Statistical Analysis

We used the Dice coefficient and area under the receiver operating characteristic curve (AUC) for evaluating agreement in segmentations, as well as the Pearson correlation coefficient (*r*) and mean absolute error (MAE) for segmentation-derived choroid thickness and area. The calculation of thickness and area from the segmentation is described in more detail in Burke et al.^20^ Briefly, for thickness, the average of three measures was used, taken at the fovea and 2000 µm from it in either direction by drawing a perpendicular line from the upper boundary to the lower boundary to account for choroidal curvature. For area, pixels were counted in a region of interest with radius 3000 µm around the fovea, corresponding to the commonly used Early Treatment Diabetic Retinopathy Study (ETDRS) macular area of 6000 × 6000 µm.^33^

We compared the agreement of DeepGPET with GPET segmentations against the repeatability of GPET itself. The creator of GPET (J.B.) made both the original and repeated segmentations with GPET. Because both segmentations were done by the same person there was no interrater subjectivity at play. Thus, the intrarater agreement measured here is a best-case scenario and forms an upper bound for agreement with the original segmentations and any other semiautomatic method requiring manual input, which can necessarily be subject to human variability, unlike DeepGPET.

In addition to quantitative evaluations, we also compared segmentations by GPET and DeepGPET for 20 test-set OCT images qualitatively by having them rated by an experienced clinical ophthalmologist (I.J.C.M.). We selected seven examples with the highest disagreement in thickness and area, seven examples with disagreement closest to the median, and six examples with the lowest disagreement. Thus, these 20 examples cover cases where both methods are very different, cases of typical disagreement, and cases where both methods are very similar. In each instance, the ophthalmologist (I.J.C.M.) was shown the segmentations of both methods overlaid on the OCT (blinded to which method produced which segmentation) and was also provided with the raw, full-resolution OCT. He was then asked to rate each one along three dimensions: quality of the upper boundary, quality of the lower boundary and overall smoothness using an ordinal scale of “very bad”, “bad”, “okay”, “good”, or “very good.”

## Results

### Quantitative

Table 2 shows the results for DeepGPET and a repeat GPET compared to the initial GPET segmentation as “ground truth.”

**Table 2.** Metrics for DeepGPET and Repeated GPET Using the Initial GPET Annotation as Ground Truth

				Thickness		Area	
Method	AUC	Dice	Time (s/image), Mean ± SD	Pearson's *r*	MAE (µm)	Pearson's *r*	MAE (mm^2^)
DeepGPET	0.9994	0.9664	1.25 ± 0.10	0.8908	13.3086	0.9082	0.0699
Repeat GPET	0.9812	0.9672	34.49 ± 15.09	0.9527	10.4074	0.9726	0.0486

#### Agreement in Segmentation

Both methods show excellent agreement with the original segmentations. The agreement of DeepGPET is comparable to the repeatability of GPET itself, with the DeepGPET AUC being slightly higher (0.9994 vs. 0.9812) and the Dice coefficient slightly lower (0.9664 vs. 0.9672). DeepGPET performing better in terms of AUC but worse in terms of Dice suggests that, for pixels where it disagreed with GPET after thresholding, the confidence is lower than for ones where it agreed with GPET. This in turn suggests that DeepGPET is well calibrated based on the raw predictions made for each pixel.

#### Processing Speed and Manual Interventions

Both methods were compared on the same standard laptop CPU. DeepGPET processed the images in only 3.6% of the time that GPET required. DeepGPET was fully automatic, and it successfully segmented all images, whereas GPET required 1.27 manual interventions on average, including selecting initial pixels and manual adjustment of GPET parameters when the initial segmentation failed.

This faster processing results in massive time savings. A standard OCT volume scan consists of 61 B-scans. With GPET, processing such a volume for a single eye takes about 35 minutes, during which a person has to select initial pixels to guide tracing (for all images) and adjust parameters if GPET initially failed (for about 25% of images). In contrast, DeepGPET could do the same processing in about 76 seconds on the same hardware, during which no manual input is needed. DeepGPET could even be GPU accelerated to cut the processing time by another order of magnitude.

The lack of manual interventions required by DeepGPET means that no subjectivity is introduced, unlike GPET, particularly when used by different people. Additionally, DeepGPET does not require specifically trained analysts and could be used fully automatically in clinical practice.

#### Agreement in Choroid Area and Thickness

**Figure 2.**
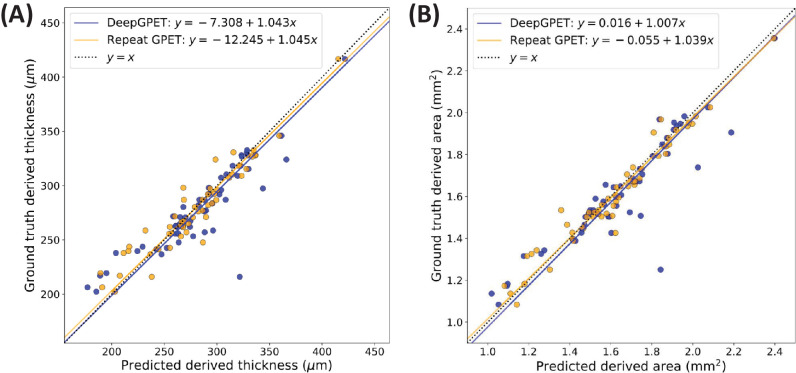
Correlation plots comparing derived measures of mean choroid thickness (**A**) and choroid area (**B**) using DeepGPET and the resegmentations using GPET.

GPET showed very high repeatability for thickness (Pearson’s *r* = 0.9527; MAE = 10.4074 µm) and area (Pearson’s *r* = 0.9726; MAE = 0.0486 mm^2^). DeepGPET achieved slightly lower, yet also very high agreement for both thickness (Pearson’s *r* = 0.8908; MAE = 13.3086 µm) and area (Pearson’s *r* = 0.9082; MAE = 0.0699 mm^2^). Figure 2 shows correlation plots for thickness and area. The agreement between DeepGPET and GPET did not quite reach the repeatability of GPET itself when used by the same experienced analyst, but it was quite comparable and high in absolute terms. Especially noteworthy is that the MAE for thickness and area was only 21% lower for thickness and 30% lower for area for repeated GPET than for DeepGPET. Thus, DeepGPET comes quite close to optimal performance (i.e., best-case repeatability where the same experienced analyst did both sets of annotation).

Furthermore, the regression fits in both derived measures for DeepGPET are closer to the identity line than for the repeated GPET measurements. For choroid thickness (CT), the linear fit estimated a slope value of 1.043 (95% confidence interval [CI], 0.895–1.192) and intercept of −7.308 µm (95% CI, −48.967 to 34.350). For choroid area (CA), the linear fit estimated a slope value of 1.01 (95% CI, 0.878–1.137) and an intercept of 0.016 mm^2^ (95% CI, −0.195 to 0.226). All CIs contain 1 and 0 for the slope and intercepts, respectively, suggesting no systematic bias or proportional difference between GPET and DeepGPET.^34^^,^^35^

**Figure 3.**
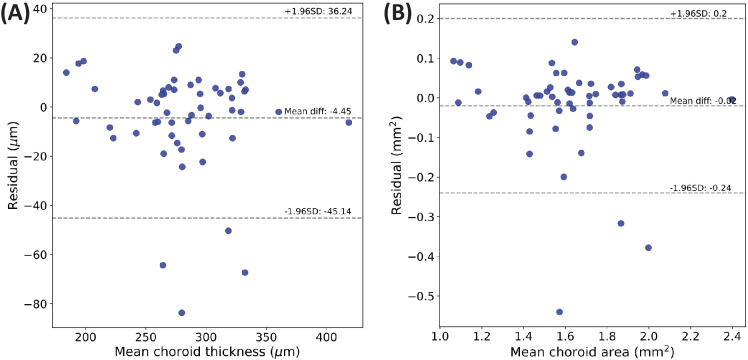
Bland–Altman plots comparing the agreement between DeepGPET and GPET using mean choroid thickness (**A**) and choroid area (**B**).

Figure 3 shows the residuals between DeepGPET and the ground-truth labels from the held-out test set using Bland–Altman plots.^36^ Rahman et al.^37^ found that intrarater agreement and interrater agreement of subfoveal choroidal thickness measurements were 23 µm and 32 µm, respectively. For CT, only 9.3% were greater than 23 µm in absolute value (5/54), with four of these representing major sources of disagreement. Similarly, for CA, the majority of residuals were centered on 0 (mean residual of −0.02 mm^2^), with only 5.5% of residuals (3/54) lying outside the limits of agreement.

### Qualitative

Table 3 shows the results of the adjudication between GPET and DeepGPET. The upper boundary was rated as “very good” for both methods in all 20 cases. However, for the lower boundary, DeepGPET was rated as “bad” in two cases for the lower boundary and one case for smoothness. Otherwise, both methods performed very similarly.

**Table 3.** Qualitative Ratings of 20 Test Set Segmentations Along Three Key Dimensions

Method	Upper Boundary	Lower Boundary	Smoothness
DeepGPET	Very good: 20	Very good: 4; good: 10; okay: 4; Bad: 2	Very good: 5; good: 12; okay: 2; bad: 1
GPET	Very good: 20	Very good: 6; good: 6; okay: 8; Bad: 0	Very good: 6; good: 13; okay: 1; bad: 0

The rater was blinded to the identity of the methods, and their order was randomized for every example.

**Figure 4.**
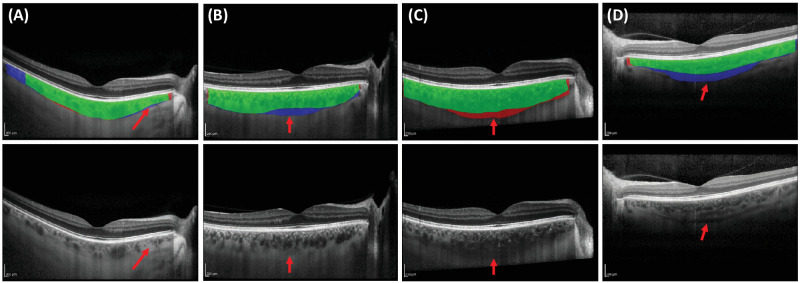
Four examples from the adjudication. The rater preferred DeepGPET for panels (**A**) and (**B**) and GPET for panels (**C**) and (**D**). (*Top row*) *Green* indicates segmentation by both GPET and DeepGPET; *red*, by GPET only; and *blue*, by DeepGPET only. (*Bottom row*) The *arrows* indicate important choroidal features that can make segmentation challenging. (**A**) No large vessels are in the nasal region to guide segmentation. (**B**) Lower boundary is very faint and below the posterior most vessels. (**C**) Lower boundary is noisy and faint. (**D**) Large suprachoroidal space is visible.

Figure 4 shows some examples. Figure 4A shows that DeepGPET segmented more of the temporal region than did GPET, thus providing a full-width segmentation that was preferred by the rater. Additionally, both approaches are able to segment a smooth boundary, even in regions with stroma fluid obscuring the lower boundary (red arrow). In Figure 4B, the lower boundary for this choroid is very faint and is actually below the majority of the vessels sitting most posterior (red arrow). DeepGPET produced a smooth and concave boundary preferred by the rater, whereas GPET fell victim to hugging the posterior-most vessels in the subfoveal region. In Figure 4C, DeepGPET rejected the true boundary in the low-contrast region (red arrow) and opted for a more well-defined one, whereas GPET segmented the more uncertain path. Because GPET permits human intervention, there is more opportunity to finetune its parameters to fit what the analyst believes is the true boundary. Here, the rater preferred GPET, whereas the under-confidence of DeepGPET led to undersegmentation and to a bad rating. In Figure 4D, the lower boundary is difficult to delineate due to a thick suprachoroidal space (red arrow) and thus a lack of lower boundary definition. Here, the rater gave a bad rating to DeepGPET and preferred GPET, but remarked that GPET actually under-segmented the choroid by intersecting through posterior vessels. The choroids in Figures 4B to 4D are the choroids with the largest CT and CA disagreement between DeepGPET and GPET as observed in Figure 3.

## Discussion

We developed DeepGPET, a fully automatic and efficient method for choroid layer segmentation, by distilling GPET, a clinically validated semiautomatic method. DeepGPET achieved excellent agreement with GPET on held-out data in terms of segmentation and derived choroidal measurements, approaching the repeatability of GPET itself and well within the threshold expected to exceed interrater agreement as observed in previous work.^37^ We also found no significant association between segmentation performance (via Dice score) and choroidal thickness or area and the Heidelberg signal-to-noise quality index in the held-out test set (Supplementary Table S3 and Supplementary Fig. S2). Most importantly, DeepGPET does not require specialist training and can process images fully automatically in a fraction of the time, enabling analysis of large-scale datasets and potential deployment in clinical practice.

Although the observed agreement was very high, it was not perfect. However, even higher agreement with GPET would not necessarily produce a better method, as GPET itself is not perfect and even conceptually there is debate around the exact location of the choroid–scleral interface (CSI), the lower choroid boundary in an OCT B-scan. CSI is commonly defined (e.g., by the original authors behind EDI-OCT^38^) as the smooth inner boundary between the choroid and sclera, or just below the most posterior vessels but excluding the suprachoroidal space. However, even that definition is still debated and can be difficult to discern in practice. Not all choroids are smooth, and there are edge cases such as vessels passing from the sclera into the choroid or stroma fluid obscurations that make the boundary even more ambiguous. These features, coupled with low signal-to-noise ratio and vessel shadowing from superficial retinal vessels, all contribute to the difficult challenge of choroid layer segmentation.

For quantitative analysis of choroidal phenotypes, the specific definition of the CSI is secondary to applying the same, consistent definition across and within patients. Here, fully automatic methods such as DeepGPET provide great benefit by removing the subjectivity present in semiautomatic methods. Where semiautomatic methods require manual input, two analysts with different understandings of the CSI could produce vastly different segmentations. With DeepGPET, the same image is always segmented in the same way, removing subjectivity.

**Figure 5.**
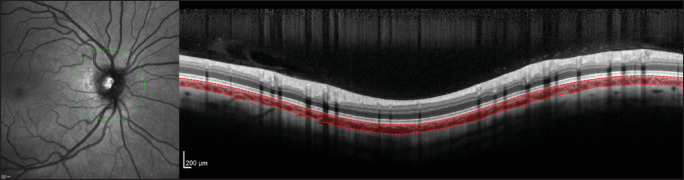
An example peripapillary scan from the Heidelberg Standard Module, automatically segmented by DeepGPET without manual intervention.

**Figure 6.**
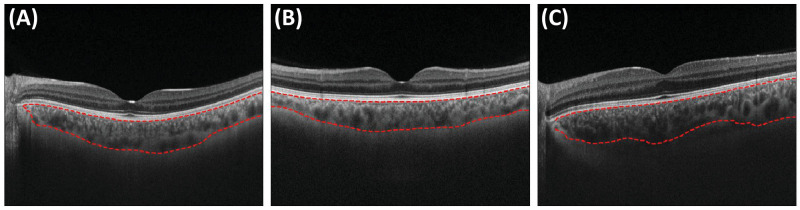
Three OCT B-scan images from a Topcon imaging device, of which two were successful (**A**, **B**) and one was not (**C**).

Initial experiments with other types of OCT imaging have positively indicated the ability of DeepGPET to generalize to different visualizations of the choroid. Figure 5 shows a peripapillary scan extracted from the Heidelberg Standard Module, centered on the optic head, with the choroid automatically segmented. Figure 6 shows choroid segmentations using DeepGPET for three OCT B-scans from a Topcon device (DRI OCT Triton Plus; Topcon, Tokyo, Japan)—two cases where DeepGPET worked well and one case where it did not. This result shows some promise in the usability of DeepGPET for scans different from the Heidelberg macular line scans on which it was trained. We hope in future iterations to extend the training data with scans from different imaging devices and scan locations. We recommend those using DeepGPET on non-Heidelberg images to review the segmentations afterward as a sanity check.

In the present work, we used data from three studies and two OCT devices and included both EDI and non-EDI scans. However, we only used data from subjects that were either healthy or had systemic but not eye disease, for which DeepGPET might not be robust. In future work, we plan to externally validate DeepGPET and include cases of ocular pathologies. A further limitation is that, although GPET has been clinically validated, not all segmentations used for training DeepGPET were entirely perfect. Thus, revisiting some of the existing segmentations and manually improving them to a “gold standard” for purposes of training the model could improve DeepGPET. For example, GPET does not always segment the entire width of the choroid. Interestingly, DeepGPET already is able to do that in some cases (Figures 4A, 5, 6), and also emulates the incomplete segmentations by GPET in other cases. A model trained on enhanced “gold standard” segmentations would produce even better segmentations.

Finally, we have focused on segmentation, as it is the most important and most time-consuming step of choroidal analysis. However, the location of the fovea on OCT images must be identified to define the region of interest for derived measurements such as thickness, area, and volume. Identifying the fovea is less time consuming or ambiguous than choroid segmentation, so we plan to extend DeepGPET to output the fovea location. This would make DeepGPET a fast and efficient end-to-end framework capable of converting a raw OCT image to a set of clinically meaningful segmentation-derived measurements. Likewise, segmenting the choroidal vessels is a very challenging task even for humans and would be prohibitively time consuming to do manually; however, in the future we aim to explore whether DeepGPET can automatically segment the vasculature within the choroid, as well.

## Conclusions

Choroid segmentation is a key step in calculating choroidal measurements such as thickness and area. Currently, this is commonly done manually, which is labor intensive and introduces subjectivity. Semiautomatic methods only partially alleviate both of these problems, and previous fully automatic methods have not been easily accessible for researchers. DeepGPET addresses this gap as a fully automatic, end-to-end algorithm that does not require manual interventions. DeepGPET provides performance similar to that of the previously clinically validated, semiautomatic GPET while being fully automatic and an order of magnitude faster. This enables the analysis of large-scale datasets and potential deployment in clinical practice without requiring a trained operator. Although the definition of the lower choroid boundary is still subject to debate (especially when it comes to suprachoroidal spaces), the most important consideration is to have a method that consistently applies the same definition across subjects and studies, which DeepGPET as a fully automatic method does. As an easily accessible, open-source algorithm for choroid segmentation, DeepGPET will enable researchers to easily calculate choroidal measurements much faster and with less subjectivity than before.

## Supplementary Material

Supplement 1

Supplement 2

Supplement 3

Supplement 4

Supplement 5
