# Supplementary material for: An Open-Source Deep Learning Algorithm for Efficient and Fully Automatic Analysis of the Choroid in Optical Coherence Tomography
Source: Transl Vis Sci Technol. 2023 Nov 21;12(11):27. doi: 10.1167/tvst.12.11.27 (PMC10668622; doi:10.1167/tvst.12.11.27)
Supplement: Supplement 1 [file tvst-12-11-27_s001.pdf]

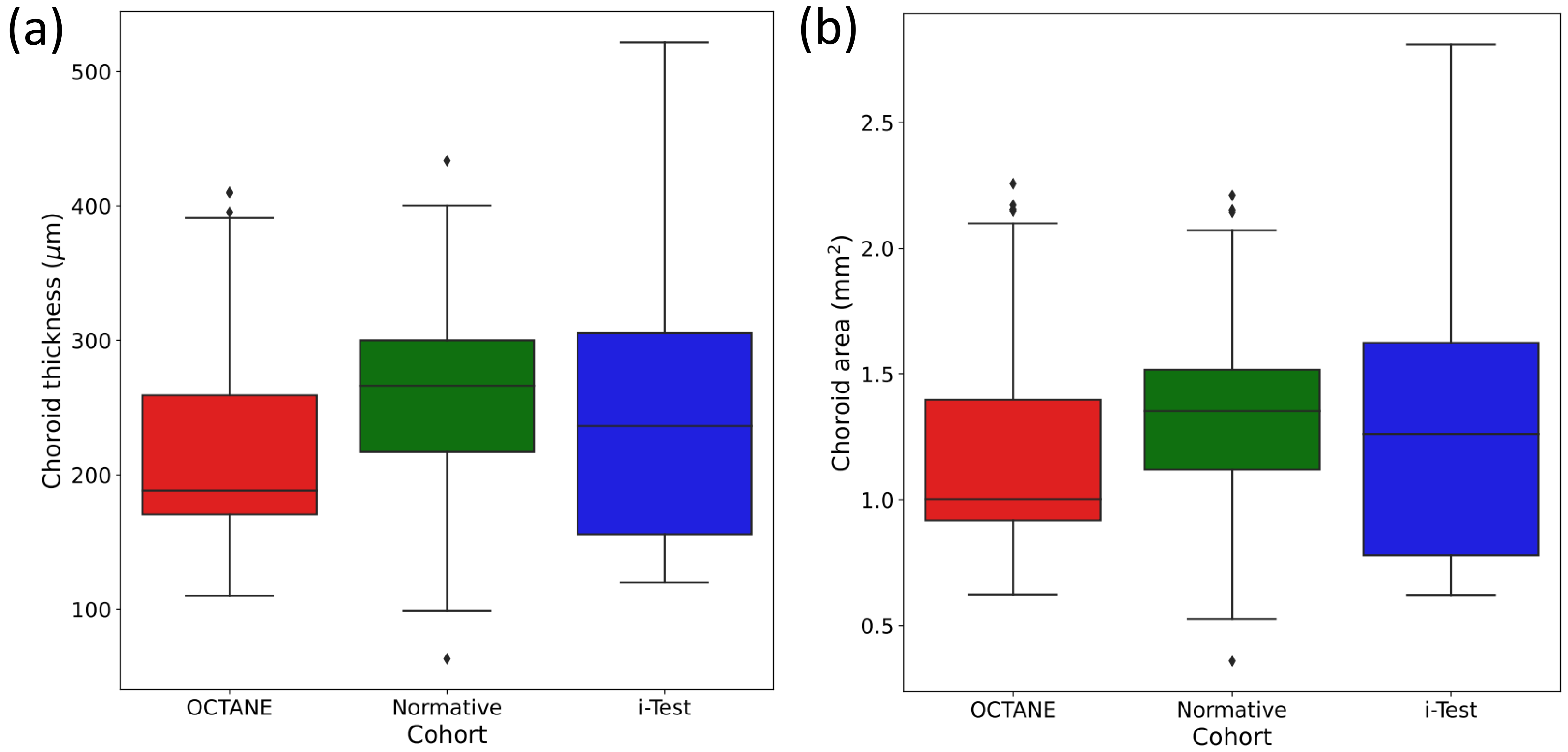

Figure S1: Box-plot distribution plots of choroid thickness (a) and choroid area (b) of the three datasets, OCTANE, i-Test and Normative.
