# Supplementary material for: An Open-Source Deep Learning Algorithm for Efficient and Fully Automatic Analysis of the Choroid in Optical Coherence Tomography
Source: Transl Vis Sci Technol. 2023 Nov 21;12(11):27. doi: 10.1167/tvst.12.11.27 (PMC10668622; doi:10.1167/tvst.12.11.27)
Supplement: Supplement 2 [file tvst-12-11-27_s002.pdf]

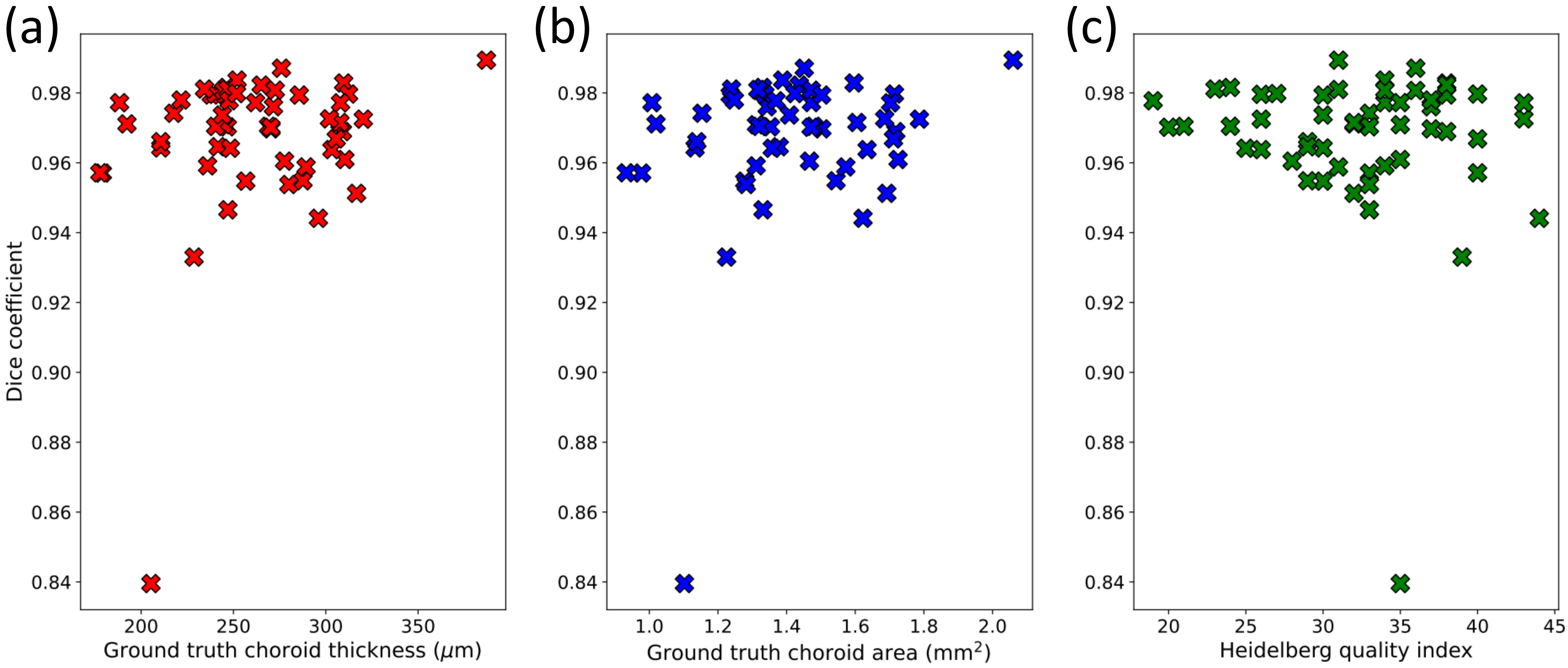

Figure S2: Test set Dice scores plotted against choroid thickness (a), choroid area (b) and Heidelberg-measured quality index (c) in the held-out test set. The outlier Dice score of approximately 0.84 is the dice score between DeepGPET and GPET from figure 4(d).
